# The anterior communicating artery variants: a meta-analysis with a proposed classification system

**DOI:** 10.1007/s00276-024-03336-7

**Published:** 2024-03-01

**Authors:** George Triantafyllou, Răzvan Costin Tudose, Christos Tsiouris, George Tsakotos, Marios Loukas, R. Shane Tubbs, Theodosis Kalamatianos, Christos Chrissicopoulos, Katerina Al-Nasraoui, Christos Koutserimpas, Mugurel Constantin Rusu, Konstantinos Natsis, Anastasios Kotrotsios, Maria Piagkou

**Affiliations:** 1 Department of Anatomy, School of Medicine, Faculty of Health Sciences, National and Kapodistrian University of Athens, 75 Mikras Asias str, Goudi, Athens, 11527 Greece; 2 Division of Anatomy, Faculty of Dentistry, “Carol Davila” University of Medicine and Pharmacy, Bucharest, Romania; 3 Department of Anatomical Sciences, St. George’s University, Grenada, USA; 4 Department of Neurosurgery, Tulane University School of Medicine, New Orleans, LA USA; 5 Department of Neurosurgery, Evangelismos Hospital, School of Medicine, Faculty of Health Sciences, National and Kapodistrian University of Athens, Athens, Greece; 6 Neurosurgery & Interventional Neuroradiology Department, Hygeia Hospital, Athens, Greece; 7 Department of Anatomy and Surgical Anatomy, School of Medicine, Faculty of Health Sciences, Aristotle University of Thessaloniki, Thessaloniki, Greece; 8 Rheumatology Clinic Iasso Thessalian Hospital, School of Medicine, University of Thessaly, Larissa, Greece

**Keywords:** Anterior communicating artery, Variation, Anatomy, Cerebral arterial circle, Aneurysm, Neuroanatomy, Variant, Morphology

## Abstract

Morphological and morphometric variants of the anterior communicating artery (AComA) have been described by multiple studies; however, a complete classification system of all possible morphological variants with their prevalence is lacking. The current systematic review with meta-analysis combines data from different databases, concerning the AComA morphological and morphometric variants (length and diameter). Emphasis was given to the related clinical implications to highlight the clinical value of their knowledge. The typical AComA morphology occurs with a pooled prevalence (PP) of 67.3%, while the PP of atypical AComA is 32.7%. The identified AComA morphological variants (artery’s hypoplasia, absence, duplication, triplication, differed shape, fenestration, and the persistence of a median artery of the corpus callosum- MACC) were classified in order of frequency. The commonest presented variants were the AComA hypoplasia (8%) and the anterior cerebral artery (ACA) fusion (5.9%), and the rarest ones were the MACC persistence (2.3%), and the AComA triplication (0.7%). The knowledge of those variants is essential, especially for neurosurgeons operating in the area. Given the high prevalence of AComA aneurysms, an adequate and complete classification of those variants is of utmost importance.

## Introduction

**Fig. 1:**
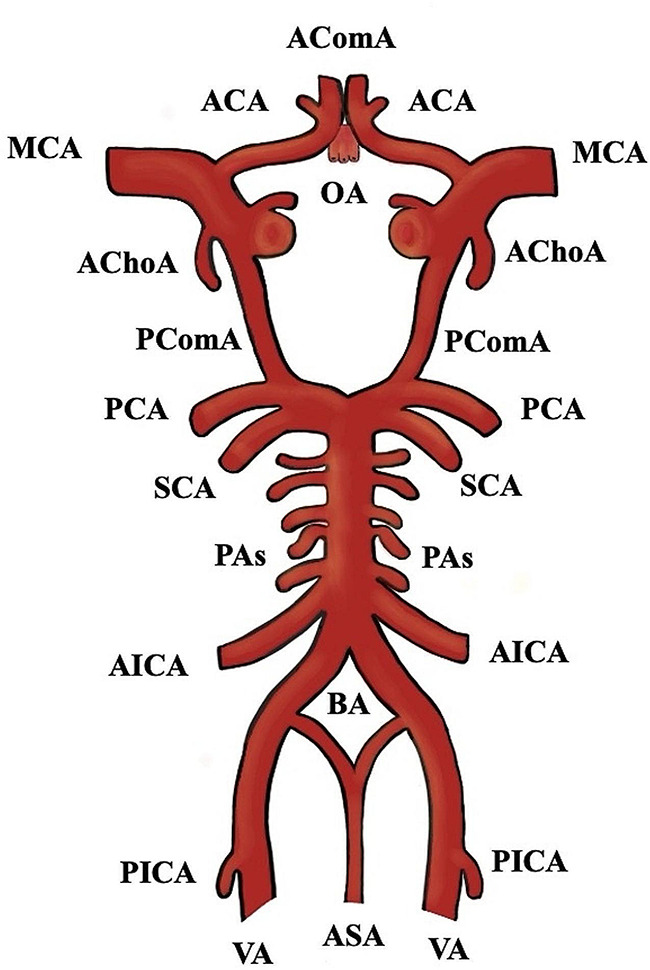
Schematic representation of the cerebral arterial circle. AComA- anterior communicating artery, ACA- anterior cerebral artery, MCA- middle cerebral artery, PCA- posterior cerebral artery, OA- ophthalmic artery, AChoA- anterior choroidal artery, PComA- posterior communicating artery, SCA- superior cerebellar artery, PAs- pontine arteries, AICA- anterior inferior cerebellar artery, BA- basilar artery, PICA- posterior inferior cerebellar artery, VA- vertebral artery, and ASA- anterior spinal artery

The cerebral arterial circle (CAC), so-called Circle of Willis, is a complex arterial network, located at the base of the brain, providing important collateral circulation to cerebral and cerebellar tissue **(**Fig. 1**).** The anterior cerebral artery (ACA) courses anteromedially above the optic chiasm and before its entrance into the interhemispheric fissure [59], joins the contralateral ACA by the anterior communicating artery (AComA). CAC variants are common (a pooled prevalence- PP of 68.22%) [24], and usually involve the anterior circulation, which is also the most frequent location of intracranial aneurysms [14]. Padget [41], first observed that the CAC variants were significantly more frequent in patients with aneurysms compared to those without. The AComA complex has a strong clinical relevance due to the common formation of intracranial aneurysms [34]. Many cadaveric and clinical studies investigated and classified the AComA variants among different populations, following different methodologies. Most of the described variants can be attributed to embryological alterations [36, 47]. The current systematic review with meta-analysis points out the AComA typical and variant morphology, highlighting common and uncommon variants, and summarizing available morphometric details of the AComA. Differences between study methods and geographic regions are further discussed.

## Materials and methods

The study was performed according to the PRISMA (Preferred Reporting Items for Systematic Reviews and Meta-Analyses) guidelines for reporting systematic reviews with meta-analyses [42].

### Search strategy

Articles were found by conducting systematic searches in the PubMed and Web of Science databases, by using the keywords “*anterior communicating artery*” AND “*anatomical variant*” AND “*morphology*” and “*anterior communicating artery*” AND “*anatomy*” AND “*morphometry*”. The reference lists of all relevant articles were thoroughly reviewed for additional relevant references to be included in the analysis. Extensive searches on Google Scholar were also conducted. Every potentially relevant article was obtained in its entirety, reviewed by two reviewers (GTr, RCT), and included if it met the inclusion criteria and did not meet any of the exclusion criteria. Articles were chosen and imported into a Paperpile database. Methods were adopted after previously published meta-analyses [20, 55].

### Selection criteria

The following exclusion criteria were used: (1) articles that did not mention any relevant measurements regarding the AComA, or presented confusing values that could not be categorized in one specific variant, (2) low-quality publications with insufficient methods for assessing and irrelevant results, or articles published in journals with a low impact factor, (3) less than 20 subjects, (4) results duplicating previously-published articles, (5) case reports, reviews, meta-analyses, or any other prevalence studies relying on published values.

### Data collection and analysis

Two reviewers (GTr, RCT) collected data from each research and stored it in separate Excel 365 databases. If a significant discrepancy arose, a third reviewer (CT) would be brought in to verify the inconsistency and determine the proper outcome. The following data was extracted: the authors’ names, the year, the technique (computed tomography-CT scan, autopsy reports, surgical reports, etc.), the total number of cases, and their evaluations.

### Quality assessment and risk of bias

For case-control studies, the Newcastle-Ottawa Scale (NOS) was used with the following modifications for a prevalence analysis: item 3 from Selection and Exposure was omitted. To measure each study’s quality, a mark ranging from 0 to 7 was assigned to it. The present paper includes articles having at least four points.

### Statistical analysis

Statistical analysis was conducted with the open-source R programming language (R Core Team, 2021) and the RStudio software (RStudio Team (2022)) using the “meta” and “metafor” packages [61, 63]. The PP for both typical and atypical anatomy variants and the pooled mean length and diameter were calculated, based on the inverse variance method and the random effects model. The proportions’ meta-analysis (prevalence meta‑analysis) was conducted using the Freeman-Tukey double arcsine transformation, the DerSimonian-Laird estimator for the between-study variance tau^2, and the Jackson method for confidence interval of tau^2 and tau. The meta-analysis of means (mean length and diameter) was conducted using the untransformed (raw) means, the restricted maximum-likelihood estimator for tau^2, and the Q-Profile method for confidence interval of tau^2 and tau [64]. The Cochran’s Q statistic was used to evaluate the heterogeneity presence across studies and the Higgins I^2 statistic was used for quantifying heterogeneity [8]. An I^2 value of 25-50%, 50-75%, and > 75% indicate low, moderate, and high heterogeneity [64, 65].

To evaluate the presence of the small-study effect (the phenomenon that smaller studies may show different effects than large ones) [60], the funnel plot asymmetry of the effect size (prevalence, length, and diameter) against the sample size was estimated by conducting regression test for funnel plot asymmetry (mixed-effects meta-regression model). Subgroup analyses were performed to estimate the impact of the study’s design (cadaveric, imaging) and subjects’ geographical region (continent of origin) on the pooled estimation. To detect outliers and influential studies the Baujat plot, the leave-one-out forest plot, and influence diagnostics were used [61, 64]. A p-value < 0.05 was considered statistically significant.

## Results

### Search synthesis

**Fig. 2:**
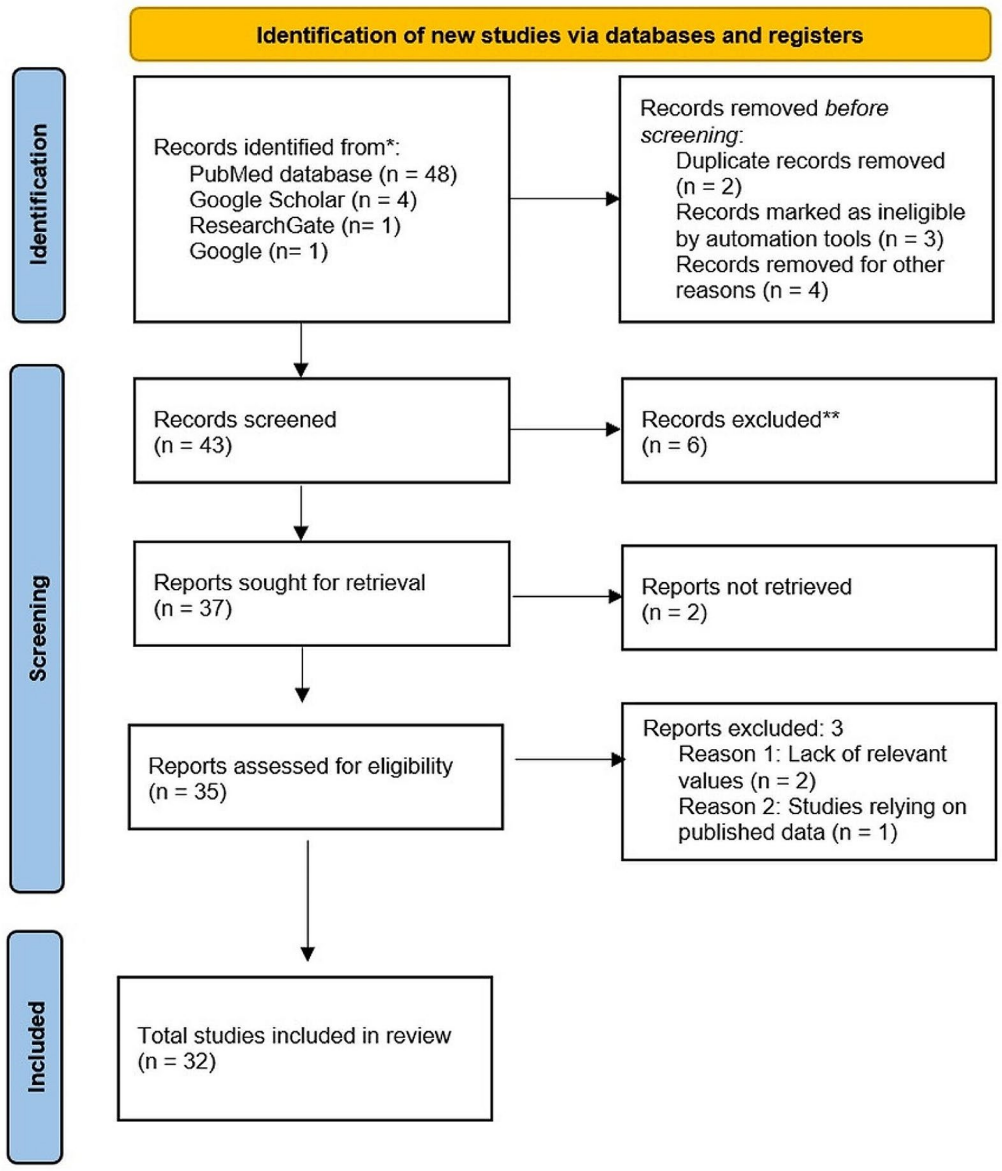
The PRISMA flow chart

During the preliminary investigation using databases and other approaches, 48 citations were acquired from PubMed, and 6 citations from the Google Scholar database. Following the exclusion of all publications that satisfied at least one exclusion condition, 32 papers were further examined and finally included in the meta-analysis. Table 1 lists each included paper. Figure 2 shows the systematized search synthesis.

**Table 1:** Studies included in the analysis. MRA-magnetic resonance angiography, CTA-computed tomography angiography, MACC- median artery of the corpus callosum, NOS- Newcastle-Ottawa Scale

Authors & Year	Study type	Number of specimens	Area of interest	NOS-modified score
Ardakani et al. (2008) [2]	Cadavers	28	Diameter	6
Blackburn et al. (1907) [3]	Cadavers	220	Typical/Absence/Fusion/Double/ Existence of MACC	5
Chen (2004) et al. [5]	MRA	507	Typical/Absence/Fusion/Double/ Existence of MACC	6
De Silva et al. (2009) [7]	Cadavers	225	Typical/Hypoplasia/Absence/Fusion/Triple/Different shaped/Existence of MACC	5
Dhanalakshmi et al. (2019) [9]	Cadavers	50	Absence/Double/Fenestration/Existence of MACC	4
Dumitrescu et al. (2022) [10]	Cadavers	96	Typical/Hypoplasia/Absence/Double/Fenestration	5
Eftekhar et al. (2006) [11]	Cadavers	102	Hypoplasia/Absence	4
Fawcett et al. (1906) [12]	Cadavers	700	Typical/Absence/Double/Triple/Existence of MACC	6
Fredon et al. (2021) [14]	MRI	669	Typical/Absence/Fusion	5
Furuichi et al. (2018) [15]	3D Reconstruction	20	Typical/Fusion/Double/Different shaped/Existence of MACC	7
Geetha et al. (2021) [16]	Cadavers	60	Typical/Hypoplasia/Absence/Fusion/Double/Triple/Different shaped/Existence of MACC	5
Hashemi et al. (2013) [18]	Cadavers	200	Typical/Hypoplasia	6
Iqbal (2013) [21]	Cadavers	50	Hypoplasia/Double/Triple/Existence of MACC	4
Jimenez-Sosa et al. (2017) [23]	CTA	283	Typical/Absence/Fusion/Double/Triple/Different shaped/Existence of MACC	6
Kamath et al. (1981) [25]	Cadavers	100	Length/Diameter	7
Kapoor et al. (2008) [26]	Cadavers	1000	Hypoplasia/Absence/Double/Triple/Different shaped/Existence of MACC	5
Karatas et al. (2015) [27]	Cadavers	100	Absence/Fusion/Double	4
Kardile et al. (2013) [28]	Cadavers	100	Hypoplasia/Absence/Fusion/Double/Triple/Different shaped/Existence of MACC	6
Krzyzewski et al. (2015) [32]	CTA	411	Typical/Hypoplasia/Absence/Fusion/Double/Existence of MACC	5
Lopez-Sala et al. (2020) [35]	CTA	426	Typical/Absence/Fusion/Double/Triple/Different shaped/Existence of MACC	6
Nyasa et al. (2021) [37]	Cadavers	24	Hypoplasia/Absence/Double	5
Ozaki et al. (1977) [40]	Cadavers	153	Typical/Absence/Fusion/Double/Triple/Different shaped/Existence of MACC	4
Puchades-Orts et al. (1976) [45]	Cadavers	62	Typical/Hypoplasia/Absence/Double/Different shaped	6
Ravikanth et al. (2019) [46]	MRA	200	Typical/Absence/Fusion/Double/Existence of MACC	5
Riveros et al. (2022) [49]	Cadavers	30	Length	4
Shatri et al. (2019) [50]	MRA	513	Typical/Hypoplasia/Fusion/Double/Fenestration/Existence of MACC	5
Stojanovic et al. (2009) [52]	Angiography	33	Typical/Fenestration/Existence of MACC	4
Thenmozhi et al. (2019) [54]	Cadavers	100	Hypoplasia/Absence/Fusion/Double/Triple/Fenestration/Existence of MACC	6
Wijesinghe et al. (2020) [56]	Cadavers	73	Length/Diameter	5
Windle (1888) [57]	Cadavers	200	Typical/Absence/Fusion/Double/Triple	7
Zurada et al. (2011) [58]	CTA	115	Length/Diameter	6

### Quality assessment and risk of bias

Each item received a score ranging from 0 to 7. Table 1 summarizes the results of each investigation. There was no evidence of substantial bias in any of the publications listed.

### Morphological parameters for AComA

#### AComA typical morphology

**Fig. 3:**
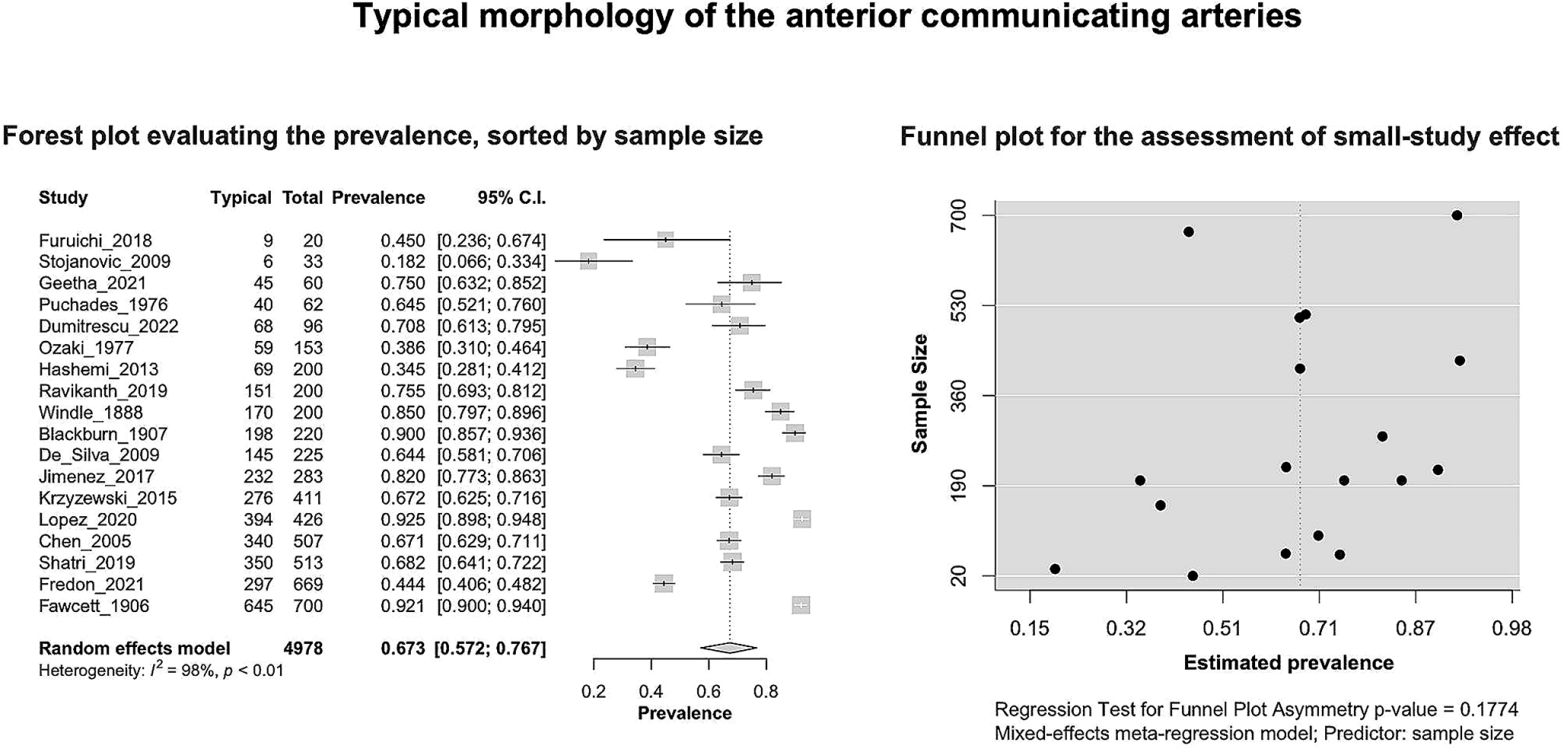
Typical morphology of the anterior communicating arteries: Forest plot evaluating the prevalence, sorted by sample size; Funnel plot for the assessment of small-study effect

**Fig. 4:**
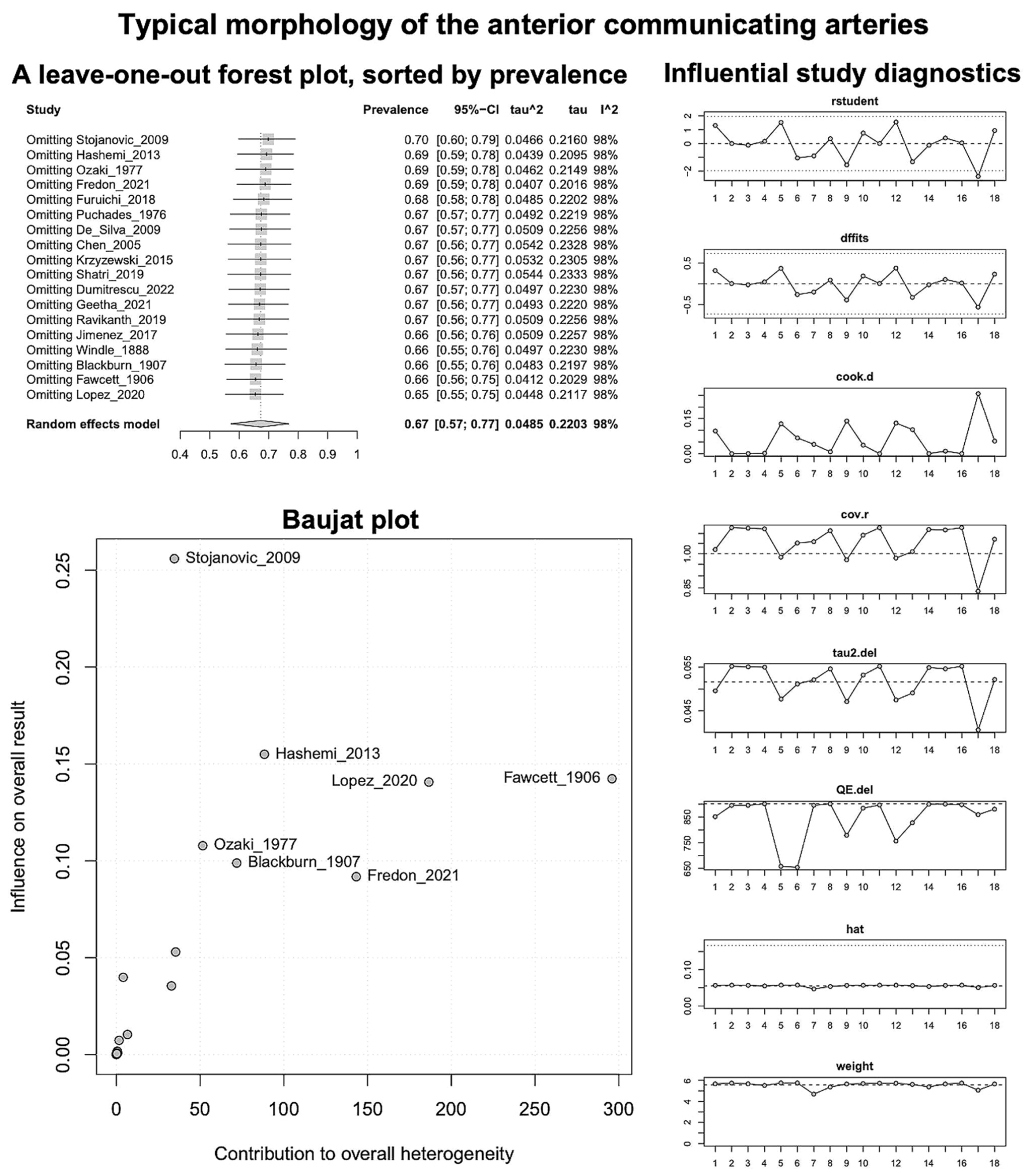
Typical morphology of the anterior communicating arteries: A leave-one-out forest plot, sorted by prevalence; Baujat plot; Influential study diagnostics

Based on the k = 18 included studies (overall cases = 4978; typical = 3494), the PP of the AComA typical morphology was estimated as 0.6732 [0.5716; 0.7674]. Approximately 67% of the population is expected to have the AComA typical morphology (Fig. 3). The estimated heterogeneity was statistically significant (*P* < 0.0001) based on the Q test statistic, and high degree based on the I^2 statistic (I^2 = 98.1%). The results of the subgroup analyses on the effect of the study’s type and the subjects’ geographical region on the estimated prevalence of the typical morphology are summarized in Table 2. The test for subgroup differences with the study’s design (cadaveric, imaging) as a categorical predictor was not statistically significant (*P* = 0.6107 > 0.05), and thus the study’s design is not a statistically significant moderator of the estimated prevalence of the typical morphology. To evaluate the geographical region as a possible moderator of the estimated prevalence of the typical morphology, the studies were categorized based on the subjects’ continent of origin. The test for subgroup differences was statistically significant (*P* = 0.0004). Only one study [23] has been included in the American subgroup, and further studies are required to reach the minimum of four studies per subgroup as suggested by Fu et al. [62] for a (categorical) subgroup variable. The estimated heterogeneity is a high degree in both Asia (I^2 = 95.2%) and Europe (I^2 = 98.8%) subgroups. Therefore, further research is required to confirm this correlation. Subsequently, the presence of the small-study effect was evaluated. The funnel plot of the prevalence against the sample size is depicted in Fig. 3. Based on the regression test, asymmetry in the funnel plot was not statistically significant (*P* = 0.1774 > 0.05) indicating no small-study effect. In addition, based on the Baujat plot and the leave-one-out forest plot (Fig. 4), no influential outlier studies (with a large impact on both the estimated PP and heterogeneity) were detected. In addition, based on the influence diagnostics results (Fig. 4), no study was identified as influential (red-colored in the diagnostics plots).

**Table 2:** The results of the subgroup analyses on the effect of the study’s design and the subjects’ geographical region on the estimated prevalence

Morphology	Moderator: Categorical predictor	Subgroups	Studies’ number (k=)	Prevalence [95%-CI]	I^2	p-value of Test for subgroup differences
Typical	Study’s design	Cadaveric	9	0.7004 [0.5292; 0.8472]	98.2%	0.6107
		Imaging	9	0.6470 [0.5158; 0.7680]	98.0%	
	Continent of origin	Asia	7	0.5789 [0.4483; 0.7042]	95.2%	**0.0004**
		Europe	8	0.6711 [0.4967; 0.8245]	98.8%	
		America	1	0.8198 [0.7727; 0.8625]	-	
Atypical	Study’s design	Cadaveric	9	0.2996 [0.1528; 0.4708]	98.2%	0.6107
		Imaging	9	0.3530 [0.2320; 0.4842]	98.0%	
	Continent of origin	Asia	7	0.4211 [0.2958; 0.5517]	95.2%	**0.0004**
		Europe	8	0.3289 [0.1755; 0.5033]	98.8%	
		America	1	0.1802 [0.1375; 0.2273]	-	
Atypical: Hypoplastic	Study’s design	Cadaveric	11	0.0829 [0.0259; 0.1649]	95.9%	0.3271
		Imaging	1	0.0535 [0.0336; 0.0776]	-	
	Continent of origin	Asia	8	0.0945 [0.0231; 0.2033]	97.0%	0.1728
		Europe	3	0.0394 [0.0133; 0.0766]	57.3%	
		Africa	1	0.1250 [0.0173; 0.2928]	-	
Atypical: Absence	Study’s design	Cadaveric	15	0.0203 [0.0069; 0.0390]	85.7%	**< 0.0001**
		Imaging	6	0.1296 [0.0860; 0.1804]	91.9%	
	Continent of origin	Asia	11	0.0383 [0.0112; 0.0783]	93.6%	**0.0006**
		Europe	6	0.0689 [0.0103; 0.1697]	98.2%	
		America	1	0.1413 [0.1030; 0.1845]	-	
		Africa	1	0.0000 [0.0000; 0.0704]	-	
Atypical: Fusion	Study’s design	Cadaveric	8	0.0576 [0.0133; 0.1269]	93.9%	0.9588
		Imaging	8	0.0592 [0.0377; 0.0848]	84.6%	
	Continent of origin	Asia	9	0.0770 [0.0365; 0.1297]	89.3%	**0.0033**
		Europe	4	0.0651 [0.0369; 0.1006]	88.3%	
		America	1	0.0177 [0.0050; 0.0370]	-	
Atypical: Double	Study’s design	Cadaveric	14	0.0655 [0.0524; 0.0800]	35.5%	**< 0.0001**
		Imaging	7	0.0085 [0.0010; 0.0206]	77.6%	
	Continent of origin	Asia	11	0.0595 [0.0308; 0.0958]	86.9%	**0.0004**
		Europe	6	0.0217 [0.0030; 0.0536]	92.7%	
		America	1	0.0035 [0.0000; 0.0151]	-	
		Africa	1	0.0417 [0.0000; 0.1702]	-	
Atypical: Triple	Study’s design	Cadaveric	9	0.0084 [0.0016; 0.0190]	69.8%	0.0976
		Imaging	2	0.0027 [0.0000; 0.0087]	0.0%	
	Continent of origin	Asia	7	0.0103 [0.0016; 0.0240]	61.0%	**0.0468**
		Europe	2	0.0017 [0.0000; 0.0054]	0.0%	
		America	1	0.0035 [0.0000; 0.0151]	-	
Atypical: Different shaped	Study’s design	Cadaveric	6	0.0541 [0.0251; 0.0922]	81.4%	0.3514
		Imaging	3	0.0269 [0.0024; 0.0685]	76.1%	
	Continent of origin	Asia	6	0.0592 [0.0252; 0.1045]	83.4%	0.2508
		America	1	0.0318 [0.0140; 0.0559]	-	
		Europe	2	0.0230 [0.0003; 0.0688]	64.6%	
Atypical: Fenestration	Study’s design	Cadaveric	3	0.0461 [0.0000; 0.1936]	92.4%	0.9662
		Imaging	2	0.0475 [0.0090; 0.1082]	49.0%	
	Continent of origin	Asia	2	0.0964 [0.0119; 0.2388]	80.2%	0.2096
		Europe	3	0.0258 [0.0000; 0.0846]	80.3%	
Atypical: MACC	Study’s design	Cadaveric	10	0.0234 [0.0126; 0.0369]	62.7%	0.8970
		Imaging	8	0.0234 [0.0124; 0.0370]	64.2%	
	Continent of origin	Asia	11	0.0227 [0.0116; 0.0367]	60.4%	0.5535
		Europe	5	0.0228 [0.0100; 0.0399]	74.7%	
		America	1	0.0389 [0.0190; 0.0650]	-	

k, Number of studies combined; 95%-CI, 95% confidence interval; I^2, Higgins I^2 statistic

#### AComA atypical morphology

**Fig. 5:**
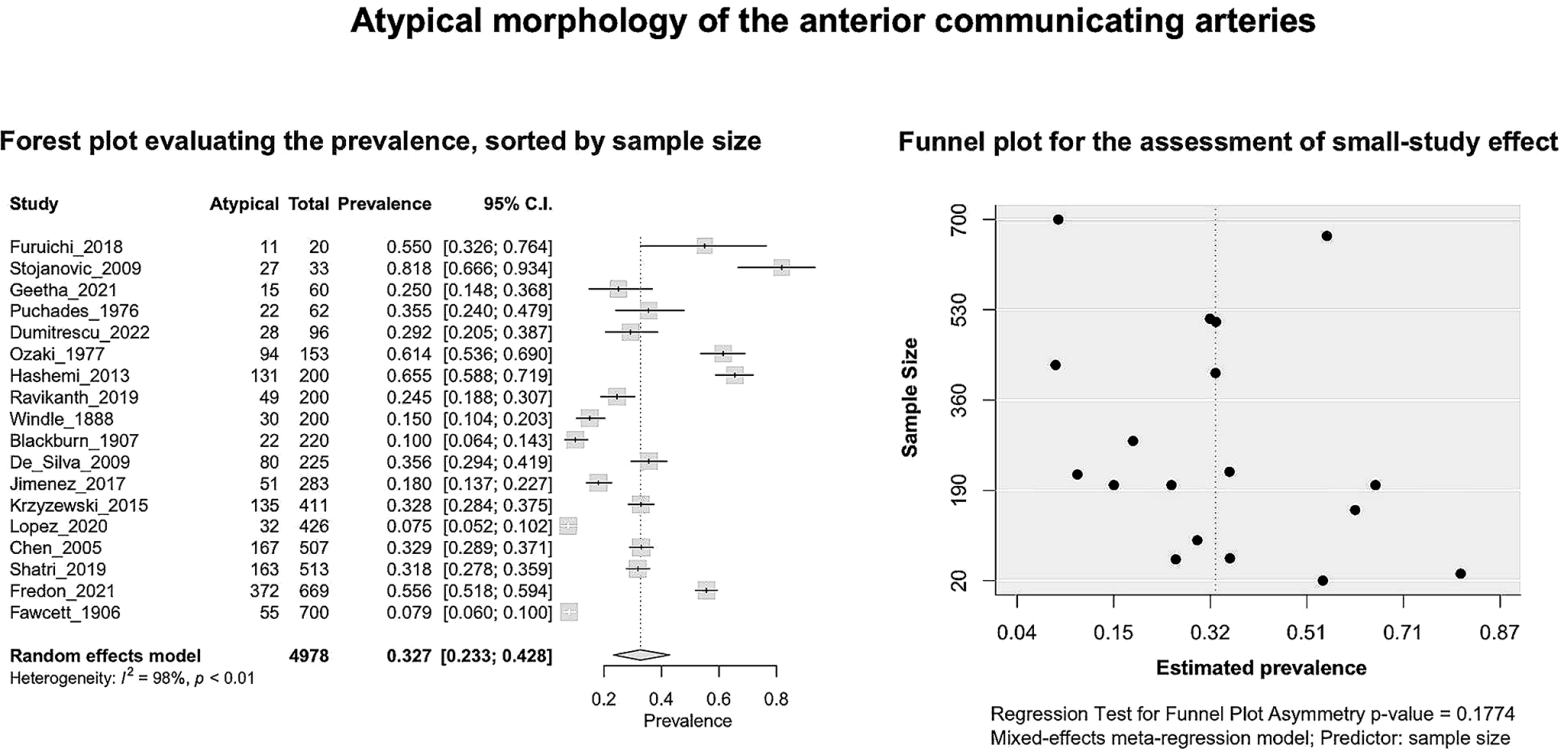
Atypical morphology of the anterior communicating arteries: Forest plot evaluating the prevalence, sorted by sample size; Funnel plot for the assessment of small-study effect

**Fig. 6:**
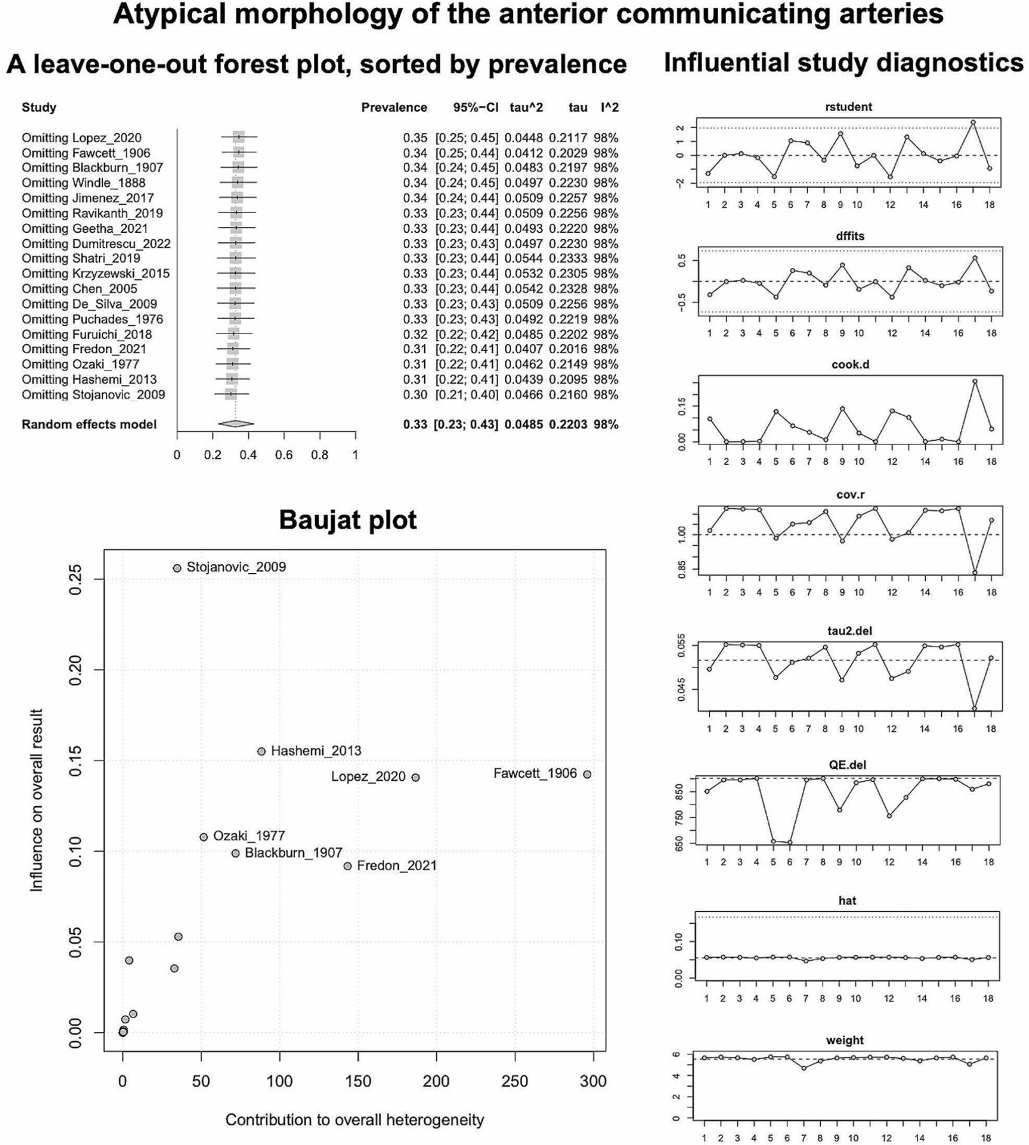
Atypical morphology of the anterior communicating arteries: A leave-one-out forest plot, sorted by prevalence; Baujat plot; Influential study diagnostics

Based on the k = 18 included studies (overall cases = 4978; atypical = 1484), the PP of the AComA atypical morphology was estimated as 0.3268 [0.2326; 0.4284]. Approximately 33% of the population is expected to have an atypical morphology (Fig. 5). The estimated heterogeneity was statistically significant (*P* < 0.0001), and high degree (I^2 = 98.1%). The subgroup analysis results on the effect of the study’s type and the subjects’ geographical region on the estimated prevalence of the atypical morphology are in line with the results for the typical morphology and summarized in Table 2. Based on the test for subgroup differences the study’s design is not a statistically significant moderator of the estimated prevalence of the atypical morphology. In addition, as reported for the typical morphology, further studies are required to confirm a possible correlation between the estimated prevalence of the atypical morphology and the geographical region. Based on the regression test for funnel plot asymmetry (Fig. 5), no small-study effect was detected. In addition, based on the Baujat plot and the leave-one-out forest plot (Fig. 6), no influential outlier studies were detected. The influence diagnostics yielded no study as influential (Fig. 6). The PP of each morphological variant was calculated:

- ***The hypoplastic AComA*** was studied in 12 articles (a total of 2430 arteries). The PP was 8% [3.1; 14.7] (Fig. 7), with significant heterogeneity (I^2^ = 95%, *P* < 0.01).
  **Fig. 7:**
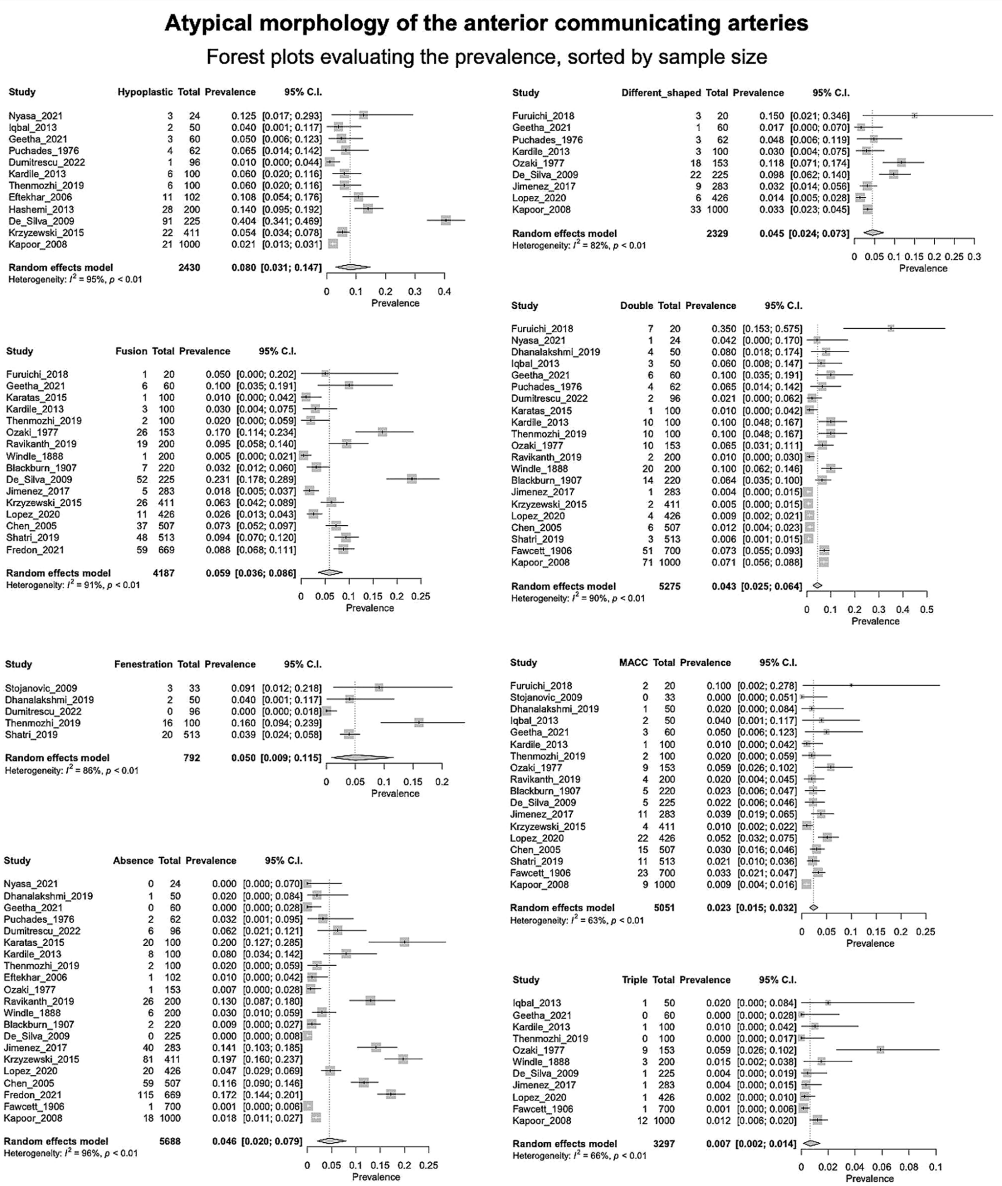
Prevalence of the anterior communicating arteries’ variants: Forest plots
- ***The fused AComA*** was encountered in 16 papers, analyzing 4187 vessels. The PP was 5.9% [3.6; 8.6] (Fig. 7), with a significant heterogeneity (I^2^ = 91%, *P* < 0.01).
- ***The AComA fenestration*** was identified in 5 papers including 792 vessels. The PP was 5% [0.9; 11.5], (Fig. 7) with a significant heterogeneity (I^2^ = 86%, *P* < 0.01).
- ***The absent AComA*** was extracted by 21 papers, adding up to 5688 arteries. The PP was 4.6% [2.0; 7.9] (Fig. 7), with a significant heterogeneity (I^2^ = 96%, (*P* < 0.001).
- ***The AComA of different shapes*** Nine articles, including 2329 vessels were included. The PP was 4.5% [2.4; 7.3] (Fig. 7). The heterogeneity was most likely significant (I^2^ = 82%, *P* < 0.01).
- ***The AComA duplication*** was identified in 21 papers, summing 5275 vessels. The PP was 4.3% [2.5; 6.4] (Fig. 7) with a significant heterogeneity (I^2^ = 90%, *P* < 0.01).
- ***The persistence of a median artery of the corpus callosum (MACC)*** was identified in 18 papers, summing 5051 arteries. The PP of the MACC persistence was 2.3% [1.5; 3.2] (Fig. 7), with a moderate heterogeneity (I^2^ = 63%, *P* < 0.01).
- ***The AComA triplication*** was found in 11 studies, which analyzed 3297 arteries. The PP was 0.7% [0.2–1.4] (Fig. 7), with moderate heterogeneity (I^2^ = 66%, *P* < 0.01).

The results of the subgroup analyses on the effect of the study’s design and the subjects’ geographical region on the estimated prevalence of each atypical morphology are summarized in Table 2. The study’s design was estimated as a statistically significant moderator of the estimated prevalence for the subgroups of the AComA absence (Cadaveric: Pr ≈ 0.0203; Imaging: Pr ≈ 0.1296; *P* < 0.0001) and double AComAs (Cadaveric: Pr ≈ 0.0655; Imaging: Pr ≈ 0.0085; *P* < 0.0001). The results indicate that the study’s design influences the estimated prevalence of both morphological variants (absent and double AComAs). Absent AComA was found to have a statistically significant higher prevalence in imaging (Pr ≈ 0.1296) than in cadaveric (Pr ≈ 0.0203) studies. The double AComA was found to have a statistically significant higher prevalence in cadaveric (Pr ≈ 0.0655) than in imaging (Pr ≈ 0.0085) studies. The subjects’ geographical region (continent of origin) was estimated as a statistically significant moderator of the estimated prevalence for the absent (*P* = 0.0006), the fused (*P* = 0.0033), the double (*P* = 0.0004) and the triple (*P* = 0.0468) AComA. However, subgroups with less than four studies have been included in the subgroup analyses (Table 2), and further studies are required to reach the minimum of four studies per subgroup as suggested by Fu et al. [62] for a (categorical) subgroup variable. Therefore, further research is required to confirm these correlations.

### Morphometrical parameters for AComA

#### AComA mean length

**Fig. 8:**
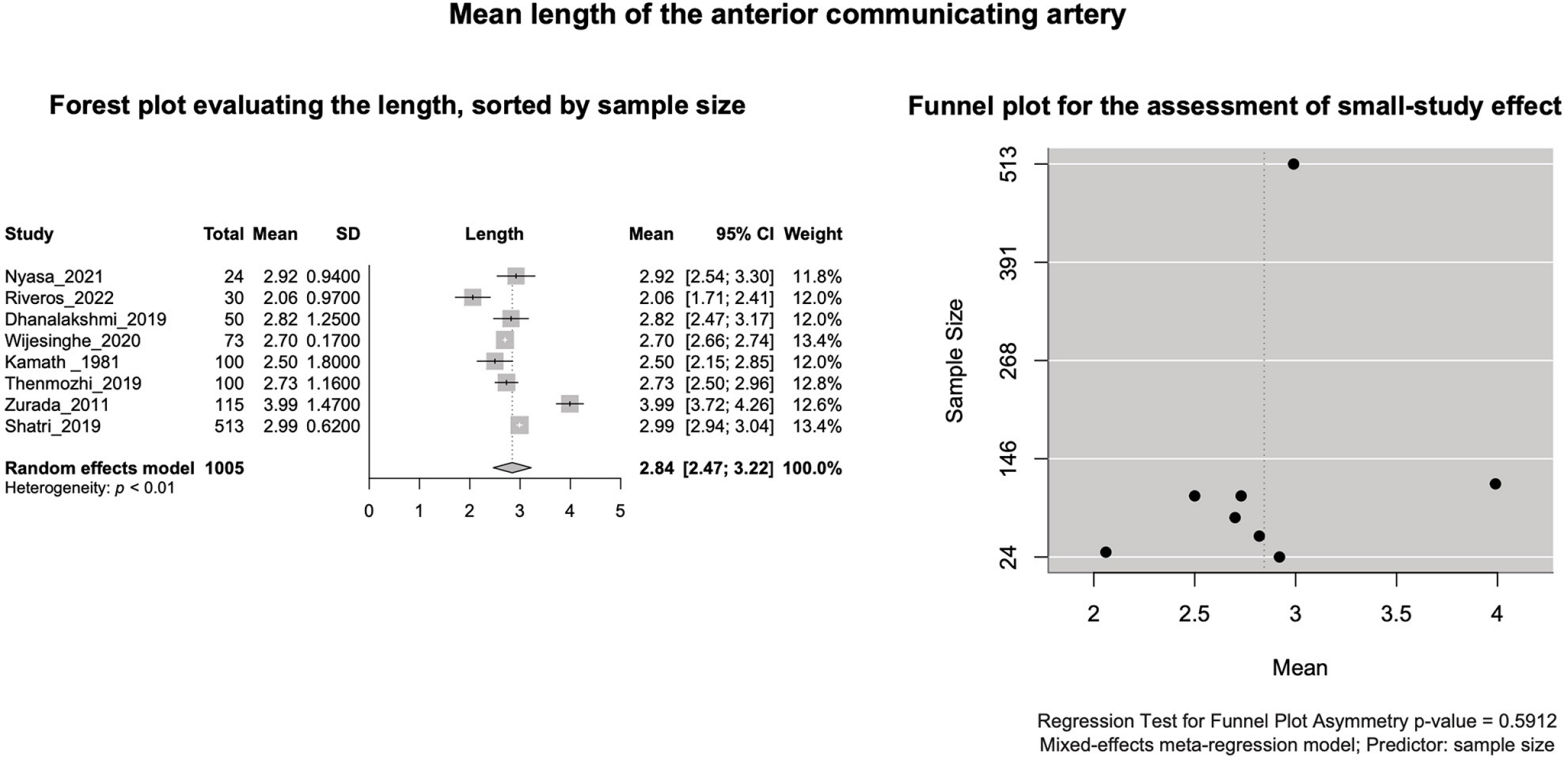
The mean length of the anterior communicating arteries: Forest plot evaluating the length, sorted by sample size; Funnel plot for the assessment of the small study effect

**Fig. 9:**
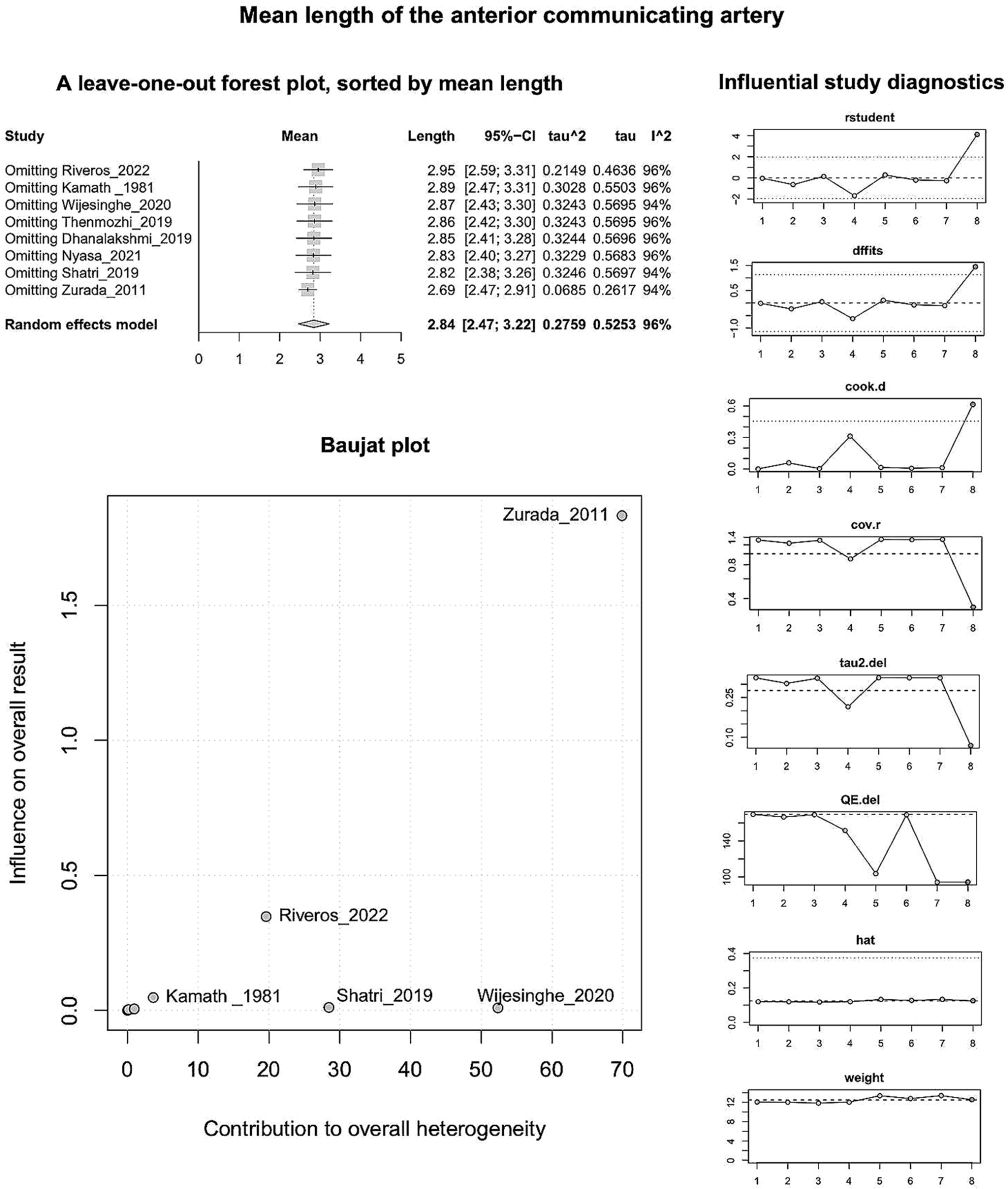
The mean length of the anterior communicating arteries: A leave-one-out forest plot, sorted by mean length; Baujat plot; Influential study diagnostics

Based on k = 8 studies (overall cases = 1005) the pooled mean length of the AComA was estimated as 2.8440 [2.4670; 3.2209] mm with statistically significant (*P* < 0.0001) and high degree (I^2 = 95.9%) heterogeneity. The forest plot evaluating the AComA pooled mean length is shown in Fig. 8. The results of the subgroup analyses on the effect of the study’s design and the subjects’ geographical region on the AComA estimated mean length are summarized in Table 3. Based on the test for subgroup differences (*P* = 0.0968 > 0.05), the study’s design (cadaveric, imaging) is not a significant moderator of the AComA estimated mean length. The subjects’ geographical region (continent of origin) was estimated as a statistically significant moderator of the estimated mean length (*P* = 0.0008). However, further studies are required to confirm this correlation to reach a minimum of four studies per subgroup [62]. The regression test for funnel plot asymmetry yielded no small-study effect (Fig. 8). Based on the Baujat plot and the leave-one-out forest plot (Fig. 9), the study conducted by Zurada et al. [58] stands out as an influential outlier by exerting substantial impact on both the estimated pooled length and heterogeneity. In addition, this study [58] was identified as influential (red-colored in the diagnostics plots) by the influence diagnostics (Fig. 9). After the exclusion of the aforementioned study, the pooled mean length was estimated as 2.6932 [2.4750; 2.9115] mm with significant (*P* < 0.0001) and high degree (I^2 = 93.6%) heterogeneity.

**Table 3:** The results of the subgroup analyses on the effect of the study’s design and the subjects’ geographical region on the estimated mean length and diameter

Morphometry	Moderator: Categorical predictor	Subgroups	k	Mean [95%-CI]	I^2	p-value of Test for subgroup differences
Length	Study’s design	Cadaveric	6	2.6300 [2.4091; 2.8510]	68.9%	0.0968
		Imaging	2	3.4810 [2.5012; 4.4608]	98.0%	
	Continent of origin	Asia	4	2.7000 [2.6620; 2.7379]	0.0%	**0.0008**
		Africa	1	2.9200 [2.5439; 3.2961]	-	
		America	1	2.0600 [1.7129; 2.4071]	-	
		Europe	2	3.4810 [2.5012; 4.4608]	98.0%	
Diameter	Study’s design	Cadaveric	6	1.4577 [1.1947; 1.7207]	96.3%	0.8911
		Imaging	2	1.5090 [0.8230; 2.1950]	99.7%	
	Continent of origin	Asia	5	1.4240 [1.1191; 1.7289]	96.6%	0.5339
		Africa	1	1.6500 [1.3940; 1.9060]	-	
		Europe	2	1.5090 [0.8230; 2.1950]	99.7%	

k, Number of studies combined; 95%-CI, 95% confidence interval; I^2, Higgins I^2 statistic

#### AComA mean diameter

**Fig. 10:**
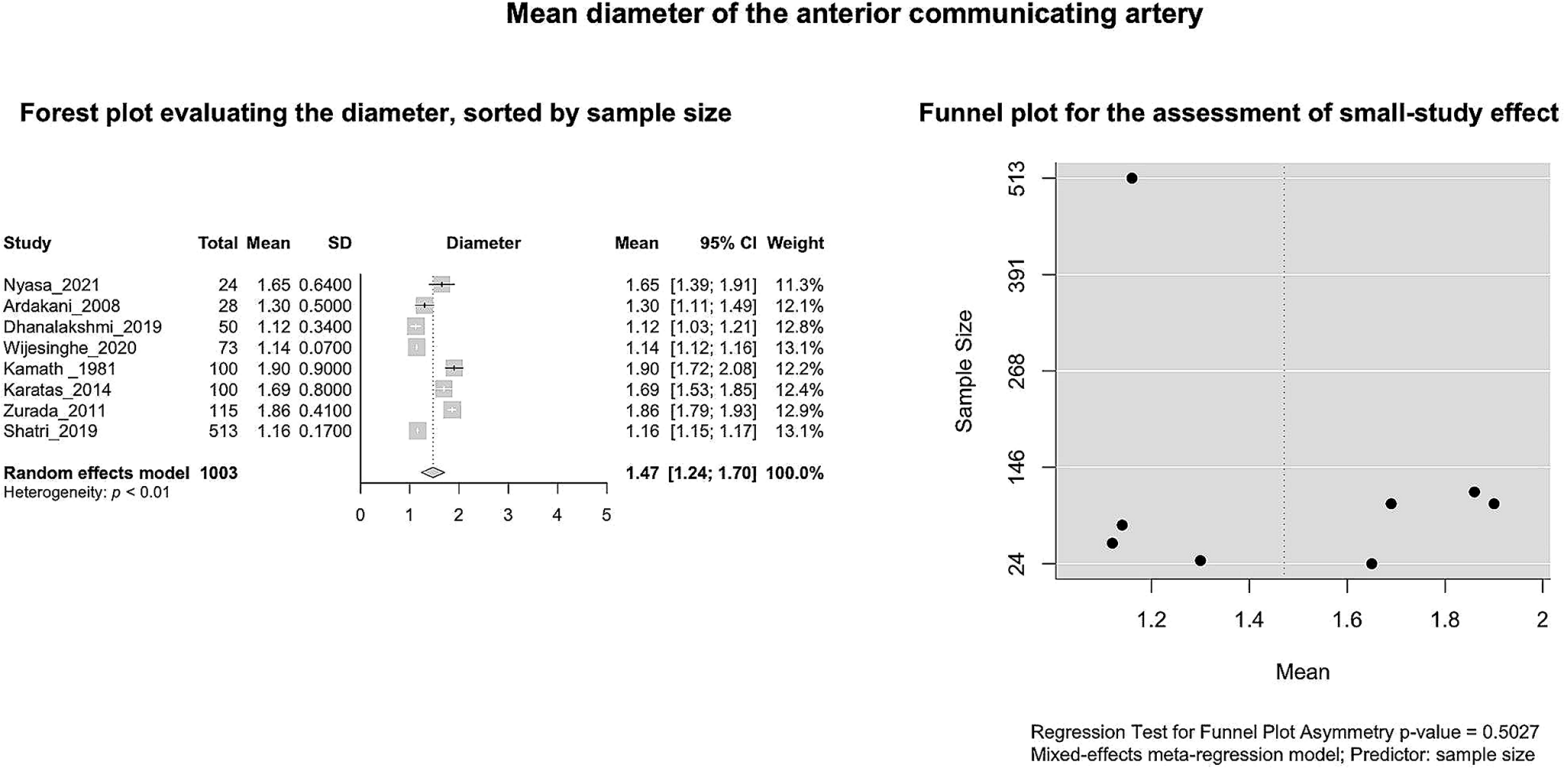
The mean diameter of the anterior communicating arteries: Forest plot evaluating the diameter, sorted by sample size; Funnel plot for the assessment of the small study effect

**Fig. 11:**
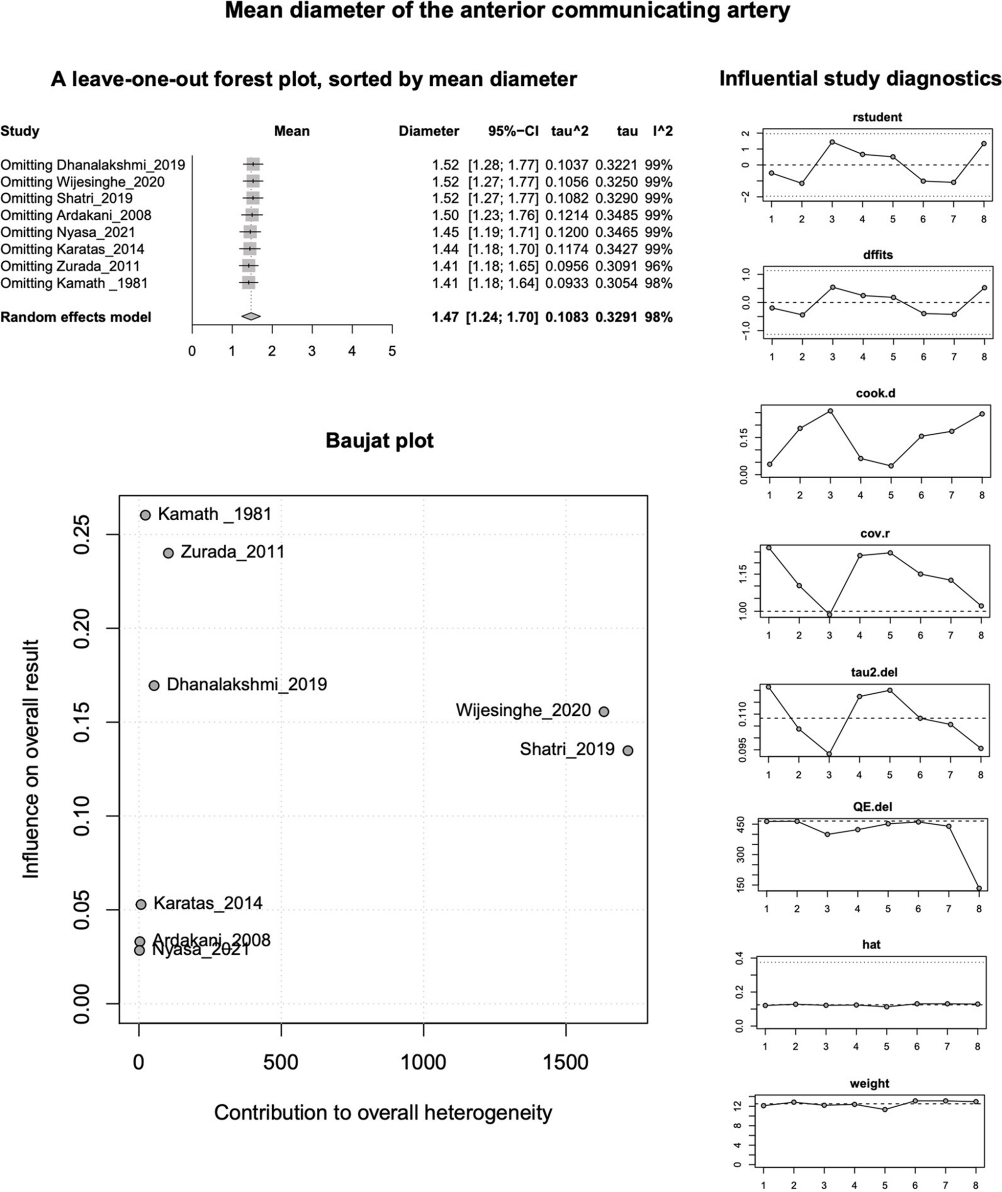
The mean diameter of the anterior communicating arteries: A leave-one-out forest plot, sorted by mean diameter; Baujat plot; Influential study diagnostics

Based on k = 8 studies (overall cases = 1003) the AComA pooled mean diameter was estimated as 1.4711 [1.2376; 1.7047] mm with significant (*P* < 0.0001) and high degree (I^2 = 98.5%) heterogeneity. The forest plot evaluating the AComA pooled mean diameter is shown in Fig. 10. The results of the subgroup analyses on the effect of the study’s design and the subjects’ geographical region on the estimated AComA mean diameter are summarized in Table 3. The test for subgroup differences yielded no significant moderator of the estimated mean diameter. The regression test for funnel plot asymmetry yielded no small-study effect (Fig. 10). Based on the Baujat plot and the leave-one-out forest plot no influential outlier studies were detected (Fig. 11). In addition, the influence diagnostics yielded no study as influential (Fig. 11).

## Discussion

### AComA morphological variants

**Fig. 12:**
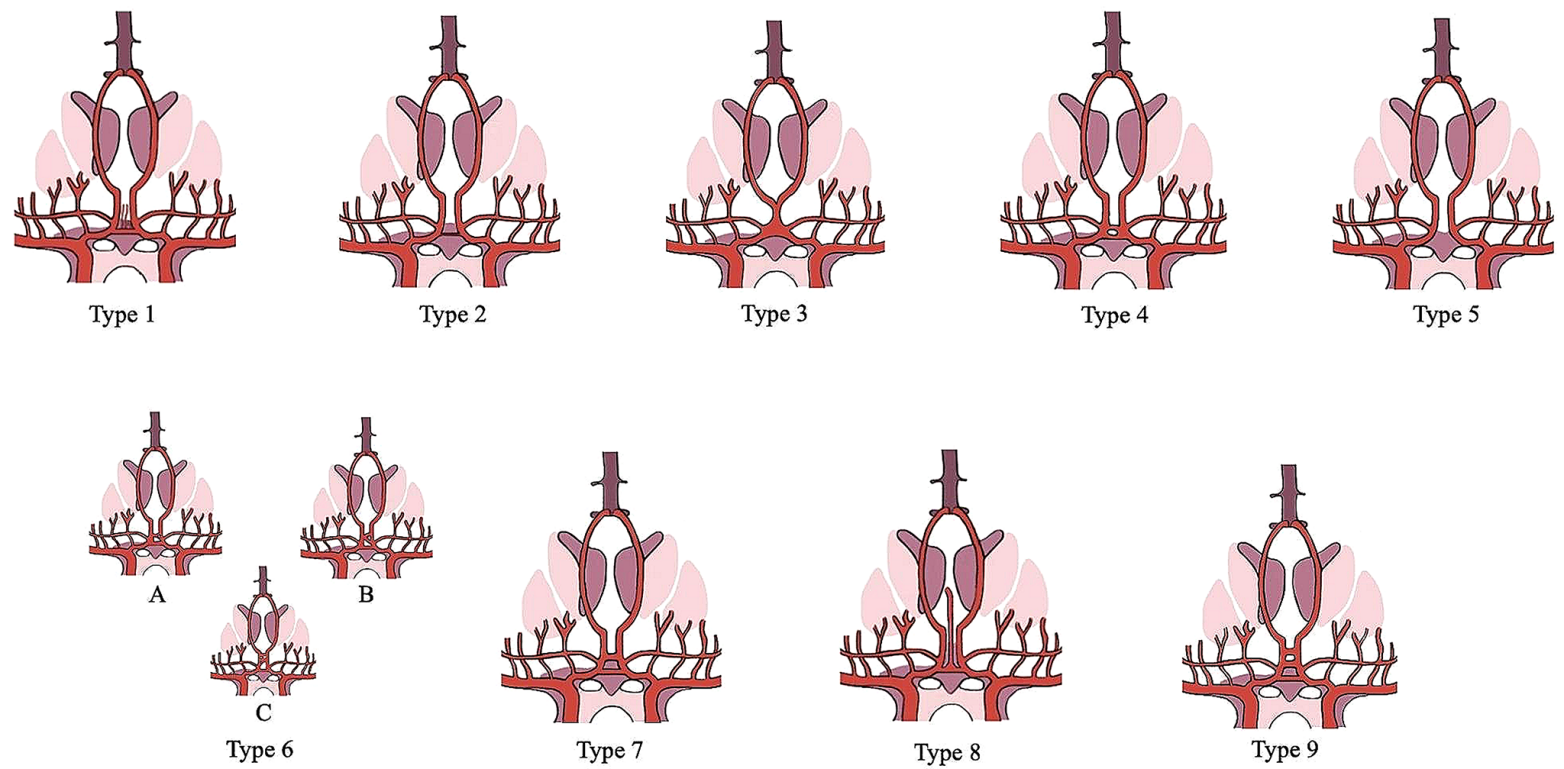
Schematic representation of the proposed classification of the anterior communicating artery (AComA) variants based on their frequency, in decreasing order. Type 1 (typical); Type 2 (hypoplastic); Type 3 (absence with ACAs fused); Type 4 (fenestration); Type 5 (absence with ACAs in parallel); Type 6 (different shaped); Type 6 A (Y-shaped); Type 6B (V-shaped); Type 6 C (plexiform); Type 7 (duplication); Type 8 (median artery of corpus collosum-MACC); Type 9 (triplication)

Despite the AComA’s great morphological variability and its clinical impact, no study summarizes results for every possible variant. The typical AComA had a 67.3% PP (Type 1), and therefore the atypical patterns occurred with 37.2% (Types 2–8). The atypical patterns will be presented with decreasing frequency. Specifically, the commonest variant was estimated as the AComA hypoplasia with an 8% PP (Type 2). Type 3 was considered the artery’s absence, where it is possible to identify two different morphological types. AComA absence with the two ACAs fused (azygos ACA) had a 5.9% PP (Type 3). Whereas AComA absence with the two ACAs coursed in parallel was estimated with a 4.6% PP (Type 5). An artery is considered fenestrated when its arterial lumen splits into two distinct channels that eventually fuse along its course [36]. Sometimes it is mistaken for duplication, which is defined as the occurrence of two distinct arteries with separate origins [36]. The AComA fenestration was estimated with a 5% PP (Type 4). The AComA could be presented in different shapes (V-shaped, Y-shaped, and plexiform). All possible shapes were considered as AComA was differently shaped with a 4.5% PP (Type 6). In addition, other rarer variants could be present. AComA duplication is estimated at a 4.3% PP (Type 7). A branch emanating directly from the AComA can be identified, which represents an embryological remnant. The MACC persistence had a 2.3% PP (Type 8). The AComA can be identified triplicated in 0.7% (Type 9). Hence, the current meta-analysis proposes a simplified classification system of the AComA morphological variability, by using the PP of its variant in decreasing order (Fig. 12). Nevertheless, it is important to mention some significant results from the meta-analysis subgroup analysis. The typical pattern had a statistically significant difference in a geographic region (America: 82%; Europe: 67%; Asia: 58%), however, only one study was included in the American region (minimum four studies per subgroup [62]). The AComA absence had an interesting statistically significant difference between the studies’ methods. The cadaveric studies’ PP was estimated at 2%, while the imaging studies had a PP of 13% (*p* < 0.0001). This relationship could be attributed to the difficulty in differentiating small arteries (small diameter and length) in imaging studies. Hence, a small AComA could be misdiagnosed as AComA absence. In cases where there is a hemodynamic imbalance between the ACAs on both sides and no flow through the AComA, it will not be visible on neuroimaging. Another significant methodological difference between the studies was identified in AComA duplication. The cadaveric studies had a 6.5% PP, while the imaging studies PP was 0.9% (*P* < 0.0001). This relationship could be attributed to the difficulty of diagnosing a second AComA that may be quite small in diameter, during an imaging study. Nevertheless, AComA absence, fusion, duplication, and triplication were identified with a statistically significant difference between geographic regions, whereas only one study was included in the American region (minimum of four studies per subgroup [62]).

### The AComA morphometric variants

Two morphometric parameters of the AComA vessels, the length, and diameter, independently affect the CAC functionality. Although the AComA morphometry is an important parameter similar to its morphology, only a few studies have investigated the AComA length and diameter. Among these studies, two unsolved questions arose:

1. What is the AComA diameter threshold to be considered hypoplastic? and.
2. What is the best method to evaluate the AComA morphometric parameters?

Generally, the blood flow volume is directly proportional to the arterial diameter and inversely proportional to the arterial length. According to Poiseulle/Hagen law: where *R* is resistance, *L* is the vessel’s length, *η* is the viscosity of the fluid (blood) and *r* is the radius of the vessel. Thus, shorter, and wider segmental arteries are favoring hemodynamics, rather than longer and narrower [37]. Another risk factor is that when AComA is longer, commonly becomes tortuous or curved [48]. For those hemodynamic reasons, it is important to investigate AComA morphometry due to the clinical implications. The current meta-analysis identified the AComA length with a pooled mean of 2.84 mm (range 0.38–10.4) and the AComA diameter with a pooled mean of 1.47 mm (range 0.2–4.9). There were no statistically significant differences between the studies’ methodology neither for the length (*P* = 0.097) nor for the diameter (*P* = 0.89). However, the results indicate a possible correlation between the studies’ method and the AComA length at the statistically significant level of 0.1 (*P* = 0.0968 < 0.1), with the estimated mean length being shorter in cadaveric studies compared to imaging studies (2.63 mm for cadaveric studies and 3.48 mm for imaging studies). This relationship could be attributed to the formalin fixation that cadaveric brains undergo, during which the vessels may change their actual size.

Among different studies, the hypoplasia diameter threshold of AComA has not been defined. Dumitrescu et al. [10] based on Iqbal [21] considered the CAC anastomotic branches (AComA and PComA) as hypoplastic when their diameter was less than 0.5 mm. Ardakani et al. [2] recorded that AComA hypoplasia is considered when the artery’s diameter is narrower than 0.6 mm. Krabbe-Hartkamp et al. [31] recommend a threshold diameter of 0.8 mm for hypoplasia, while most of the studies refer to the AComA hypoplasia narrower than 1 mm. These results’ variants could be attributed to different methods and techniques among studies (cadaveric brains, magnetic resonance, and computed tomography angiography- MRA and CTA, etc.). Cadaveric brains undergo formalin fixation and other preserving methods, during which vessels may change their actual size. Clinical imaging (MRA and CTA) studies do not identify small vessels and falsely identify them as absent (previously pointed out that AComA absence was identified statistically more frequent in imaging studies), while cadaveric studies allow direct visualization of vessels, which allows a better result [24]. Nevertheless, it is noteworthy that Jones et al. [24] in their meta-analysis did not indicate any significant differences between the findings of cadaveric and clinical studies, and the same holds for the current meta-analysis. Hence, AComA arteries narrower than 1 mm could be safely defined as hypoplastic according to most studies [11, 18, 26–28, 33, 37]. However, it is important to note that an adequate definition of AComA hypoplasia can be achieved only by hemodynamic studies investigating when a vessel diameter is narrow enough to be considered hypoplastic and cause hemodynamic dysfunction. In this context, a previous study indicated that the minimal threshold diameter for collateral flow through the CAC ranges between 0.4 and 0.6 mm; however, this study was conducted on a very small sample of 12 patients [19].

### Clinical implications of the AComA variants

The knowledge of the AComA morphological variants has clinical significance, especially for interventionists. Kayembe et al. [30] reported a clear correlation between the CAC variants and cerebral aneurysms and identified many aneurysm cases in the presence of a combination of variants. Leipzig et al. [34] studied the intraoperative aneurysm rupture on 1694 patients with intracranial aneurysms and found the AComA, the second most frequent ruptured aneurysmal artery. Patients with AComA ruptured aneurysms display damage to deep, medial frontal areas, like septal nuclei [9]. Many studies have confirmed that A1 ACA variants (especially the hypoplastic ACA) may cause AComA aneurysms due to the hemodynamic stress and the compensatory shunting of blood through AComA [13, 14, 29, 30, 33, 39, 48, 51, 52]. Rhoton [48] suggested that the greater the diameter difference between bilateral A1 segments, the more likely an AComA aneurysm will develop. Papantchev et al. [44] in a sample of 500 CAC, found that in cases of a hypoplastic or absent AComA, the left ACA was at risk of hypoperfusion during unilateral selective cerebral perfusion, a technique used for cerebral protection. According to Poiseulles-Hagen law, hypoplastic segments offer higher resistance than normal arteries. Thus, during unilateral selective perfusion, blood will follow the lowest resistance course through normal vessels and will bypass the hypoplastic ones, which will lead to hypoperfusion of certain brain areas [43]. The ACAs fusion (5.9% PP) variant and, especially, the AComA fenestration (5% PP) have been associated with aneurysm presence due to the turbulent flow created by a lack of tunica media in the proximal and distal region of the fenestration [23]. Jacquens et al. [22] were the first to associate AComA hypoplasia or absence with an increased incidence of vasospasm. Krzyzewski et al. [33] supported that AComA absence (4.6% PP) increases the risk of ischemia, as it disrupts the CAC circulation. The fact that females have more frequent AComA variants should alert neurosurgeons [33].

Currently, digital subtraction angiography (DSA) is considered the gold standard method to evaluate the CAC morphology and particularly the detection of possible areas of aneurysms [17]. When examining an AComA aneurysm, it is important to estimate the presence of a persistent MACC (2.3% PP) as this vessel becomes one of the draining arteries of the aneurysm [38]. Ogawa et al. [38] highlighted the significance of the persistent MACC coexisting with AComA aneurysm. The persistent MACC courses parallel to and posterior to the pericallosal artery, and this is why it can be easily damaged intraoperatively [38]. Precisely, they identified that in 81.5% of their studied cases, the aneurysm was formed at the AComA trifurcation, ACA A1 segment, and the MACC which makes the surgical manipulations extremely difficult [38]. In this early study (1990), it was indicated that a bifrontal craniotomy and an interhemispheric approach are the best techniques for aneurysmal surgery, due to the wide operative field and the ability to understand the AComA anatomy and adjacent structures [38]. However, nowadays, the pterional approach is popular for the treatment of AComA aneurysms [53]. Overall, it has been proved that smoking, A1 segment asymmetry, pulsatility index in the A1 segment, and the angle between the A1 and A2 segments are independent risk factors for the development of an AComA aneurysm [29]. Alfano et al. [1] observed a significant association between vessel’s bifurcation and aneurysm development (for example, Y-shaped, or V-shaped AComA) due to high wall shear stress. Even recovery levels from vascular diseases, like ischemic stroke patients, may be altered due to the CAC variants. Chuang et al. [6] identified that patients with a typical CAC have earlier improvement than patients with a variant circle in ischemic stroke patients. Especially, in the anterior brain circulation where cerebral infracts most commonly occur, variations are of great importance particularly during surgery [50]. Except for aneurysm formation, the CAC variants have been associated with mental diseases. Blackburn [3] in 220 patients with mental diseases, identified a predominance of variant circles among the mentally diseased. Blackburn [4] analyzed 42 cases of ACA fusion in a total of 400 patients with mental diseases (10.5%), they observed that fusion occurred in all forms of diseases, possibly a little more frequent in dementia. Kamath [25] reported a higher incidence of variant CAC in mentally diseased patients. As intriguing as these findings are, they were based on assumptions and are not definite.

### Limitations and future perspectives

The current review has some limitations. The sample had a great degree of heterogeneity for several reasons. There were studies with different methodologies (clinical, cadaveric studies) and different diameter thresholds to define hypoplastic AComAs. In addition, none of the studies analyzed every possible AComA variant. Hence, future studies investigating AComA morphology should use the proposed classification system to study all AComA variants properly. Nevertheless, when conducting the subgroup analysis for geographic regions, only one study was included from America. For this reason, the results could not be safely evaluated because the minimum of four studies per subgroup could not be reached [62]. Except for more systematic anatomical studies on AComA typical and variant morphology, studies investigating the clinical outcomes of patients with variable AComA, as well as the genetic basis of these variants, will further enhance our knowledge.

## Conclusion

The current systematic review with meta-analysis depicts the AComA variants and proposes a simplified classification system (based on the PP). The AComA typical pattern was identified in 67.3%, and the variant morphology was identified in 32.7%. Pitfalls associated with imaging studies can include false identification of arterial absence or duplication. Knowledge of AComA variants can aid the planning of neurosurgical procedures including those on the highly prevalent AComA aneurysms.

## Data Availability

No datasets were generated or analysed during the current study.
